# Supplementary figures and images for: METTL18-mediated histidine methylation of RPL3 modulates translation elongation for proteostasis maintenance
Source: eLife. 2022 Jun 8;11:e72780. doi: 10.7554/eLife.72780 (PMC9177149; doi:10.7554/eLife.72780)

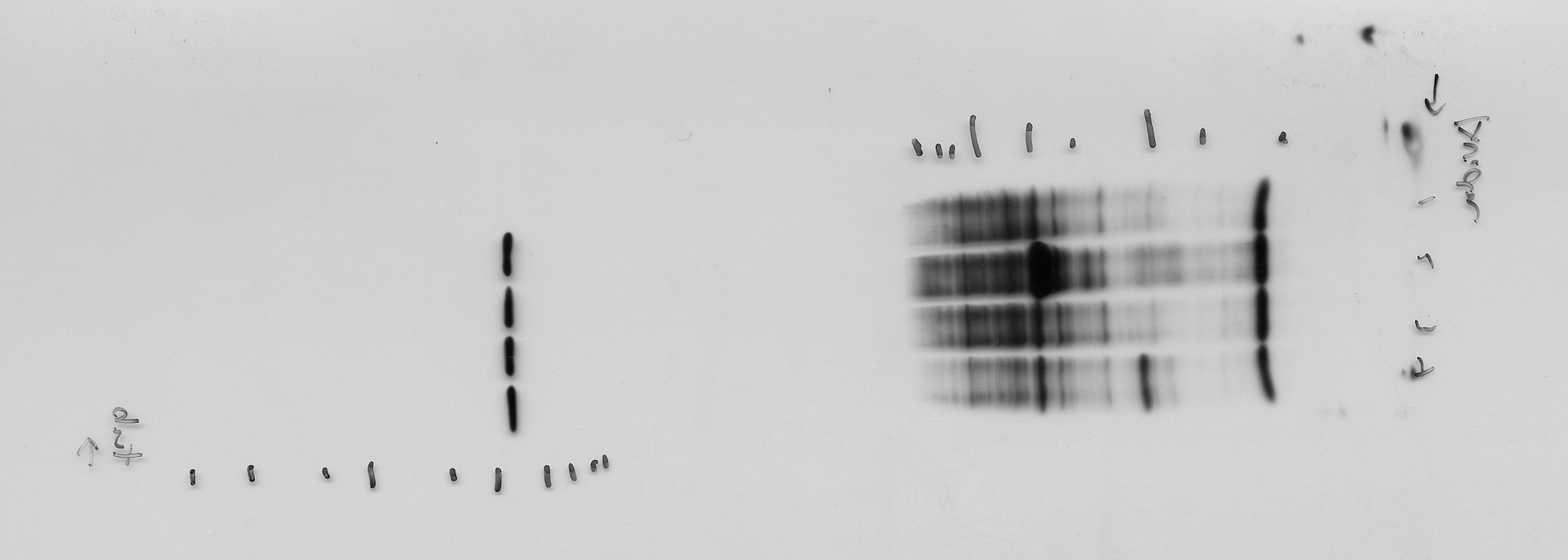

Supplement: Figure 1—source data 1. [file elife-72780-fig1-data1.tif]

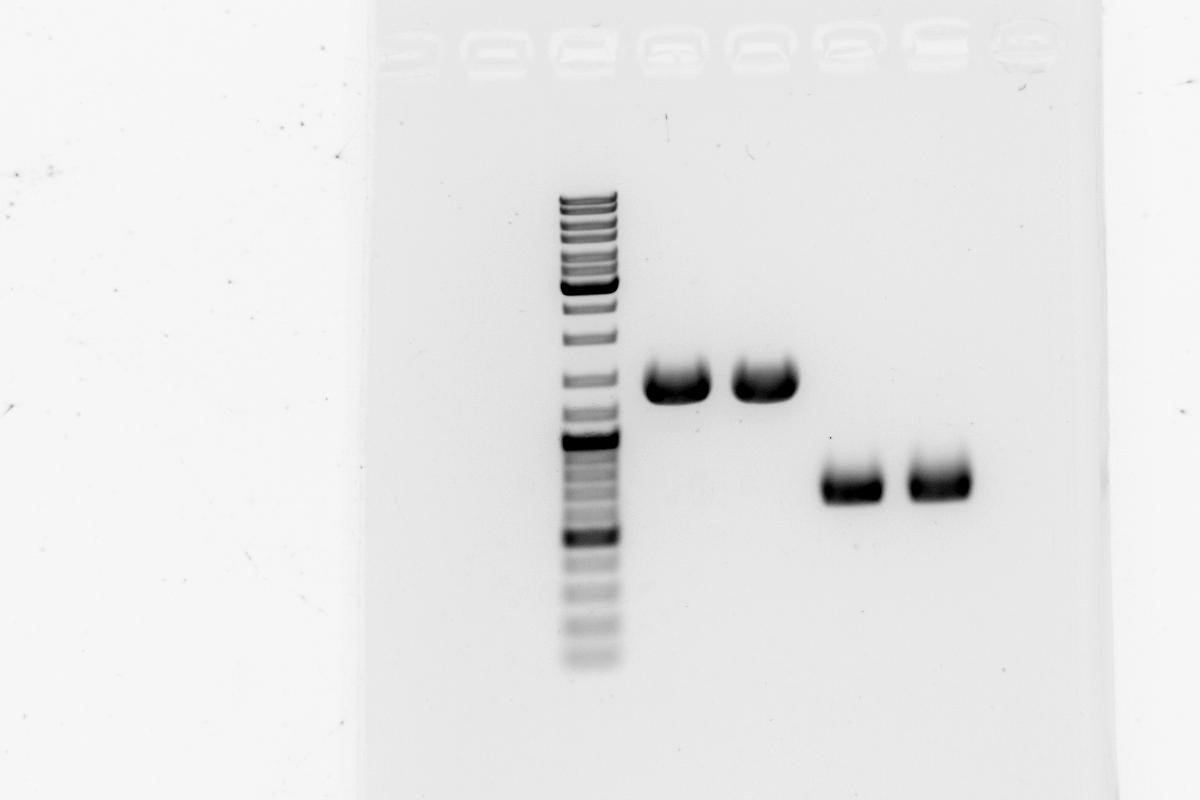

Supplement: Figure 1—figure supplement 1—source data 1. [file elife-72780-fig1-figsupp1-data1.zip › Figure1-figure supplement1C-source data1.jpg]

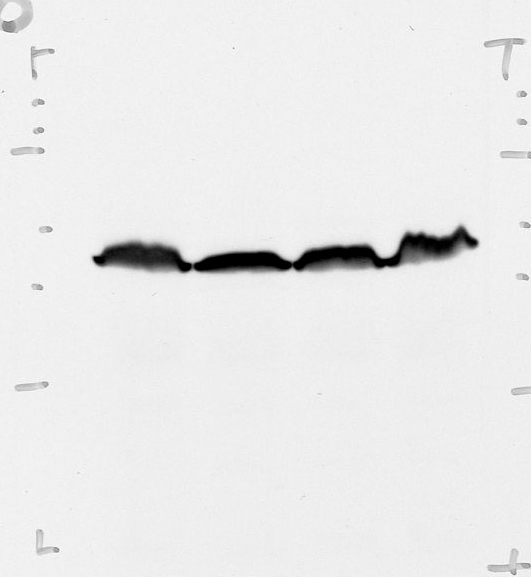

Supplement: Figure 1—figure supplement 1—source data 2. [file elife-72780-fig1-figsupp1-data2.zip › 0bc41175-5971-45b4-963c-7248c90acc60.tif]

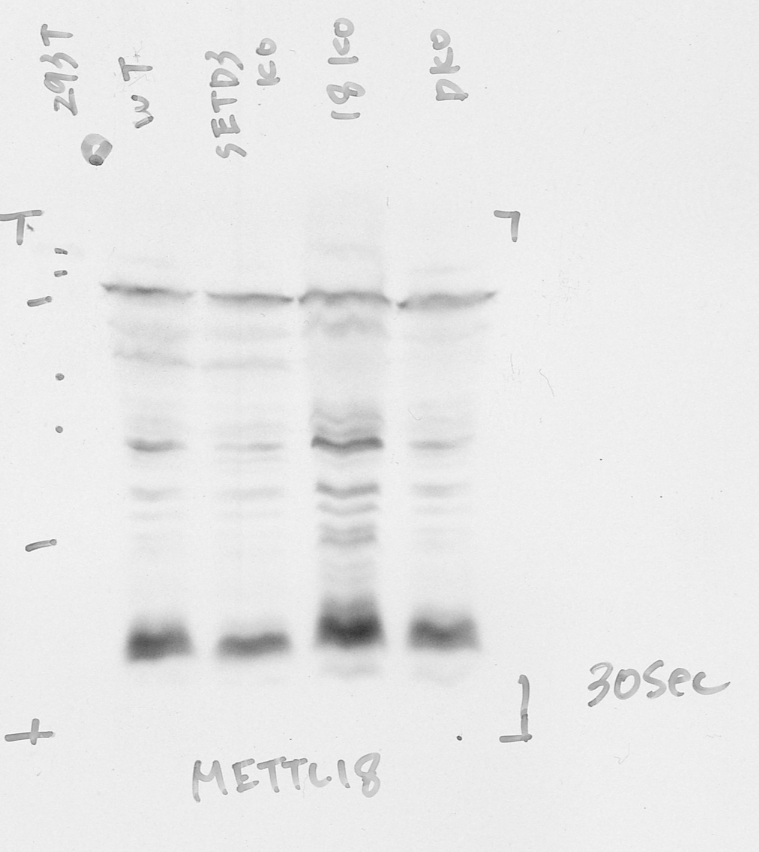

Supplement: Figure 1—figure supplement 1—source data 2. [file elife-72780-fig1-figsupp1-data2.zip › 58229011-ece2-4628-875e-a8d29af12b6b.tif]

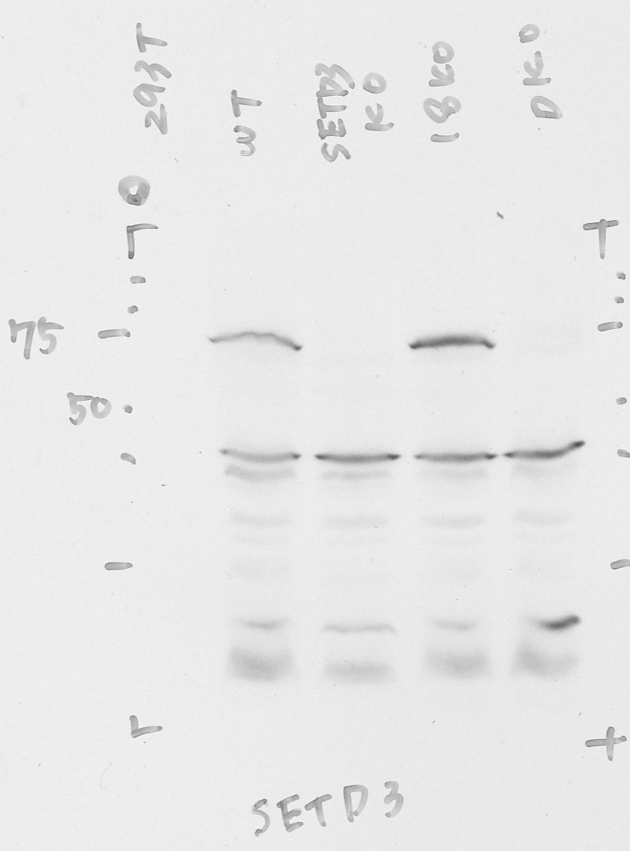

Supplement: Figure 1—figure supplement 1—source data 2. [file elife-72780-fig1-figsupp1-data2.zip › d039d750-05a4-4188-8ae4-429bb5757b67.tif]

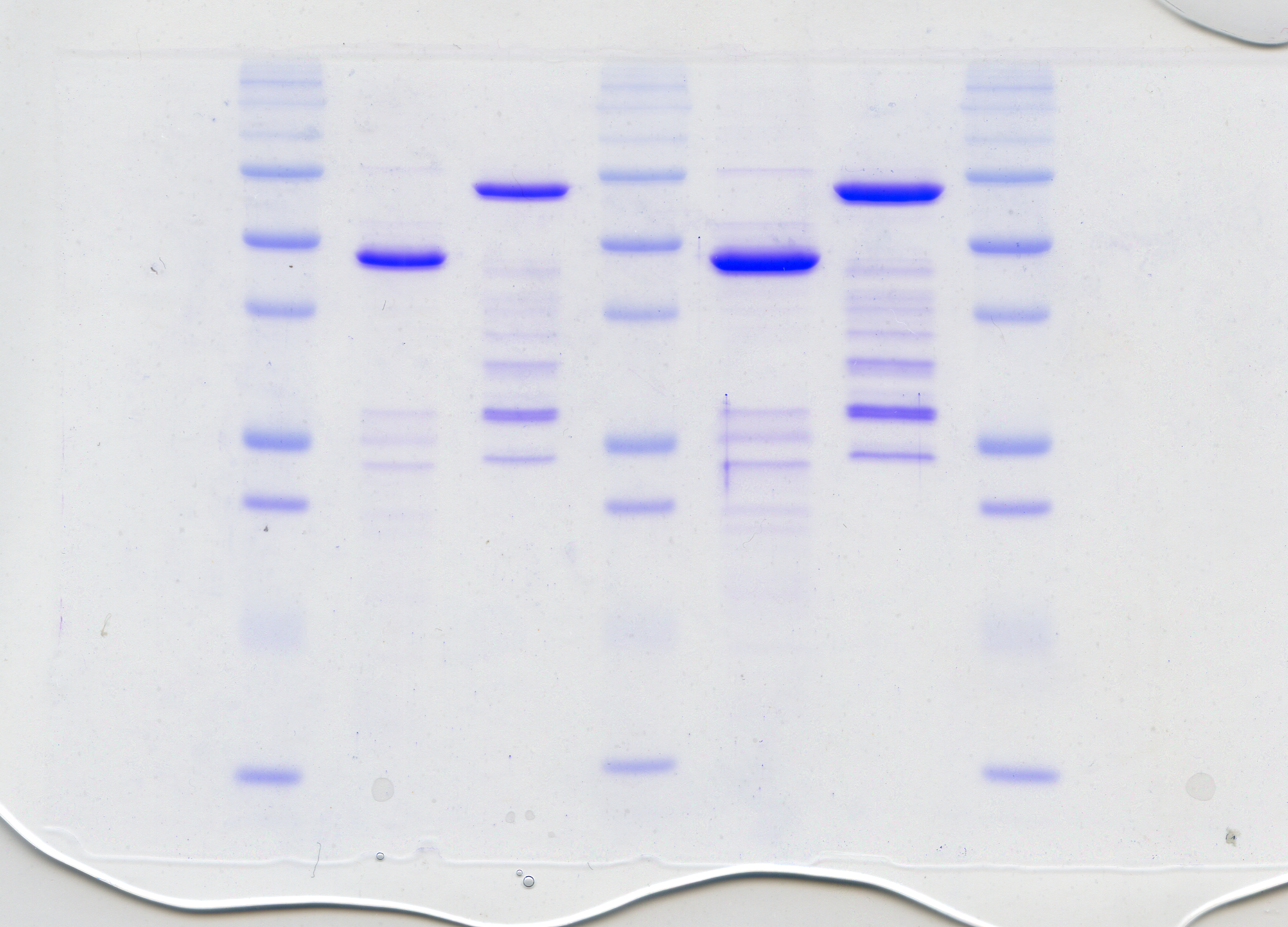

Supplement: Figure 1—figure supplement 1—source data 3. [file elife-72780-fig1-figsupp1-data3.tif]

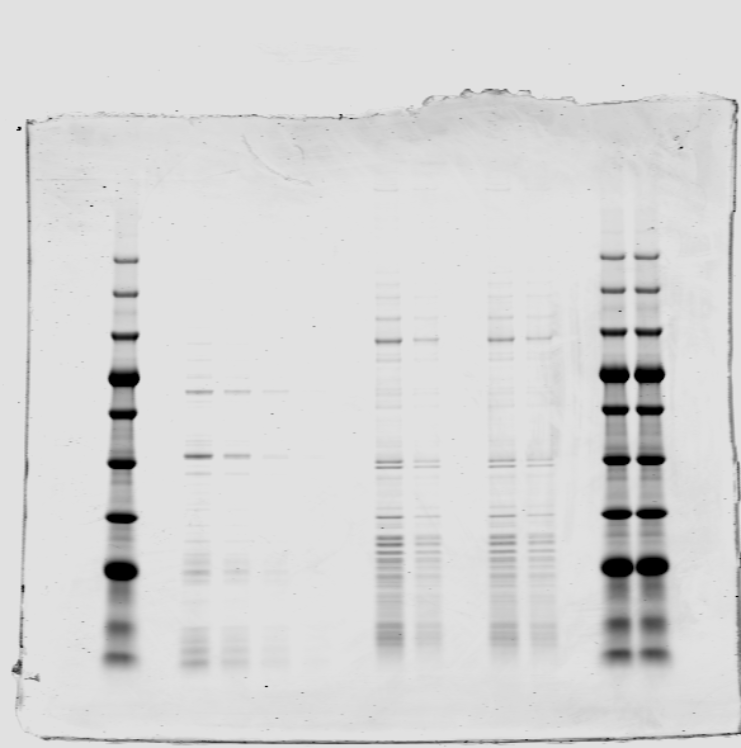

Supplement: Figure 1—figure supplement 2—source data 1. [file elife-72780-fig1-figsupp2-data1.tif]

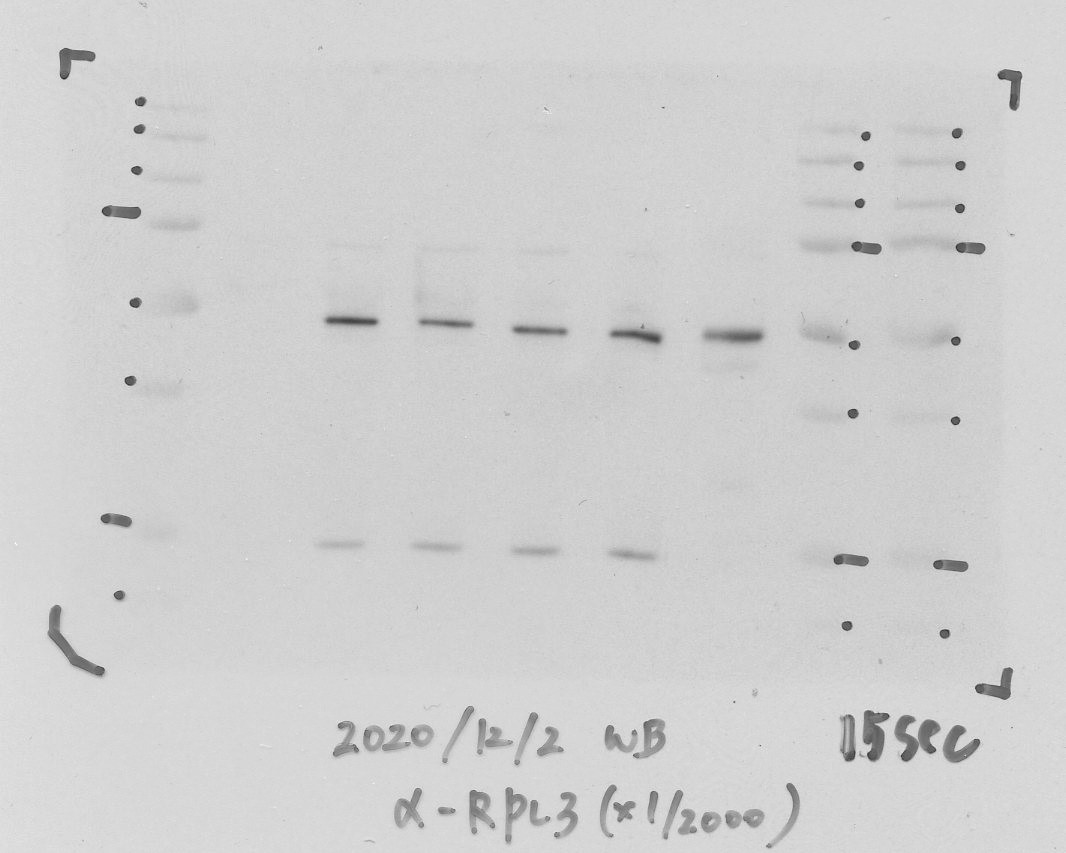

Supplement: Figure 2—source data 1. [file elife-72780-fig2-data1.zip › 0579a1f4-7163-404d-9fd5-d43475675014.jpg]

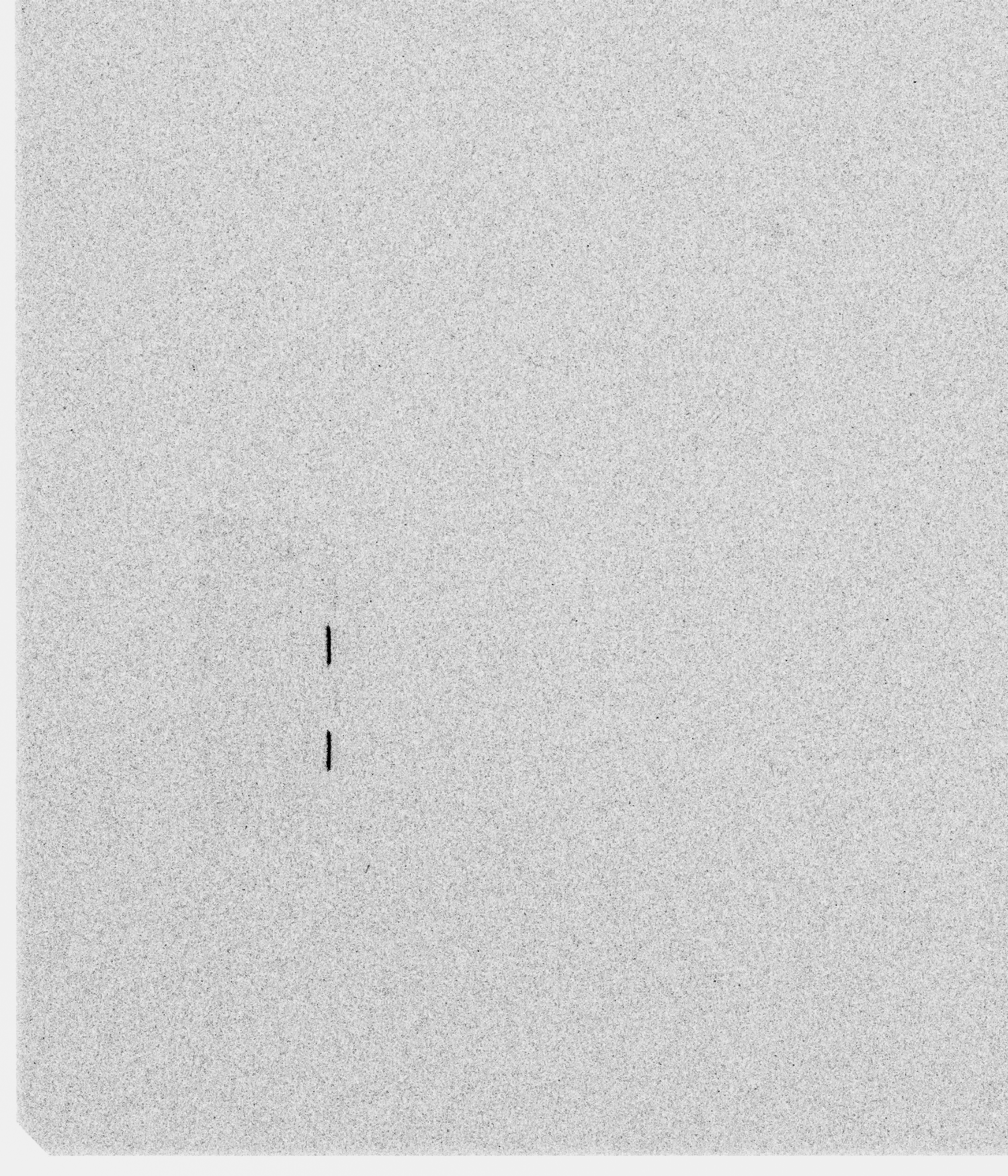

Supplement: Figure 2—source data 1. [file elife-72780-fig2-data1.zip › f3fab834-45ec-498f-a30b-263237ea20b1.tif]

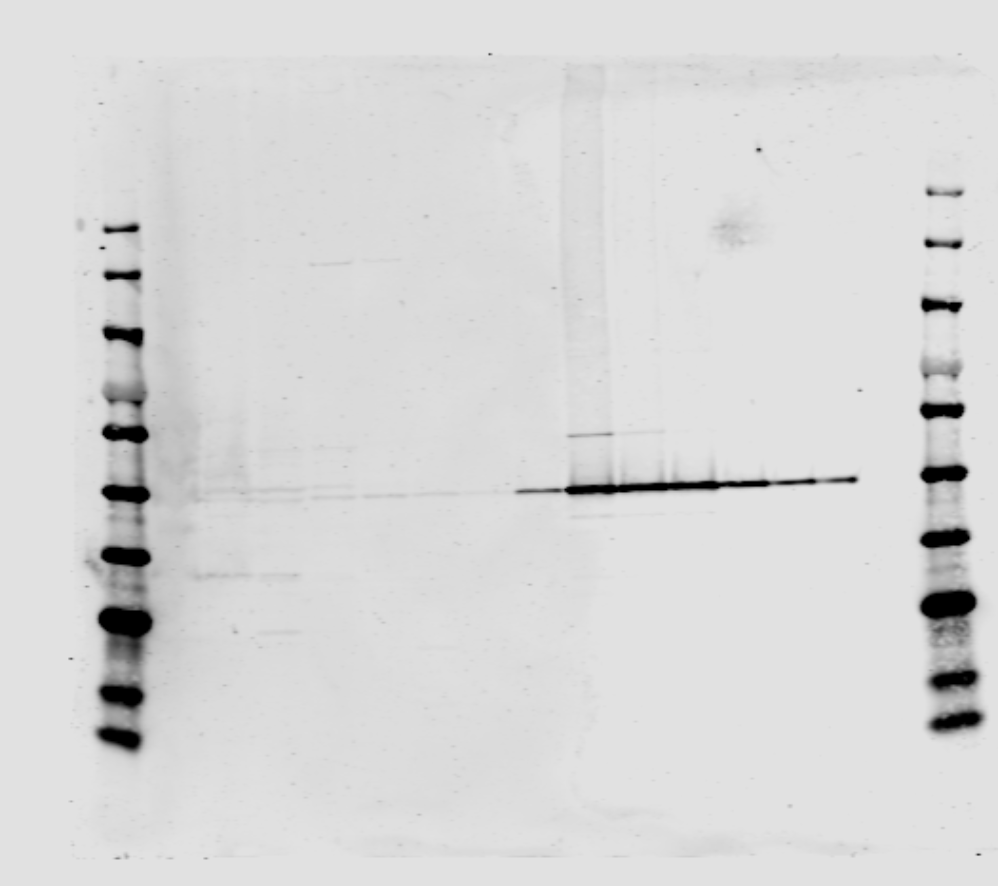

Supplement: Figure 2—source data 2. [file elife-72780-fig2-data2.zip › 25a594db-b379-413c-9c0a-65775511a8a4.tif]

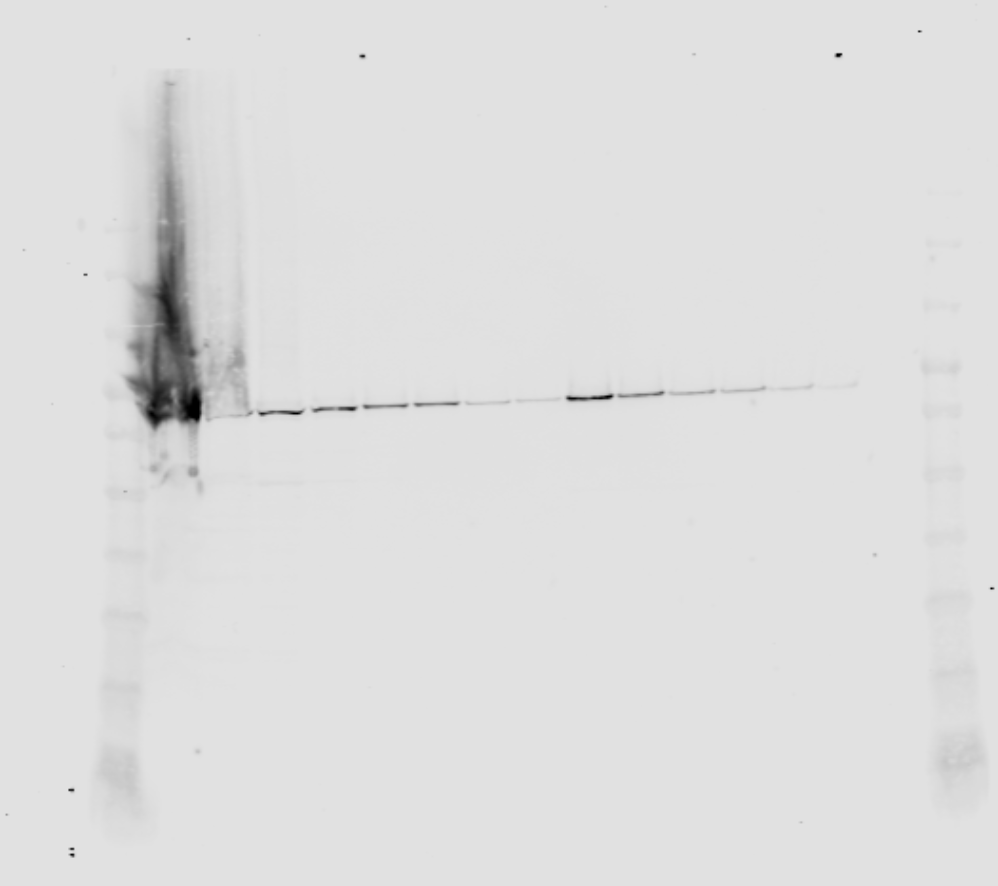

Supplement: Figure 2—source data 2. [file elife-72780-fig2-data2.zip › 8070ee9d-da49-4e53-b063-2548f9ce3a3a.tif]

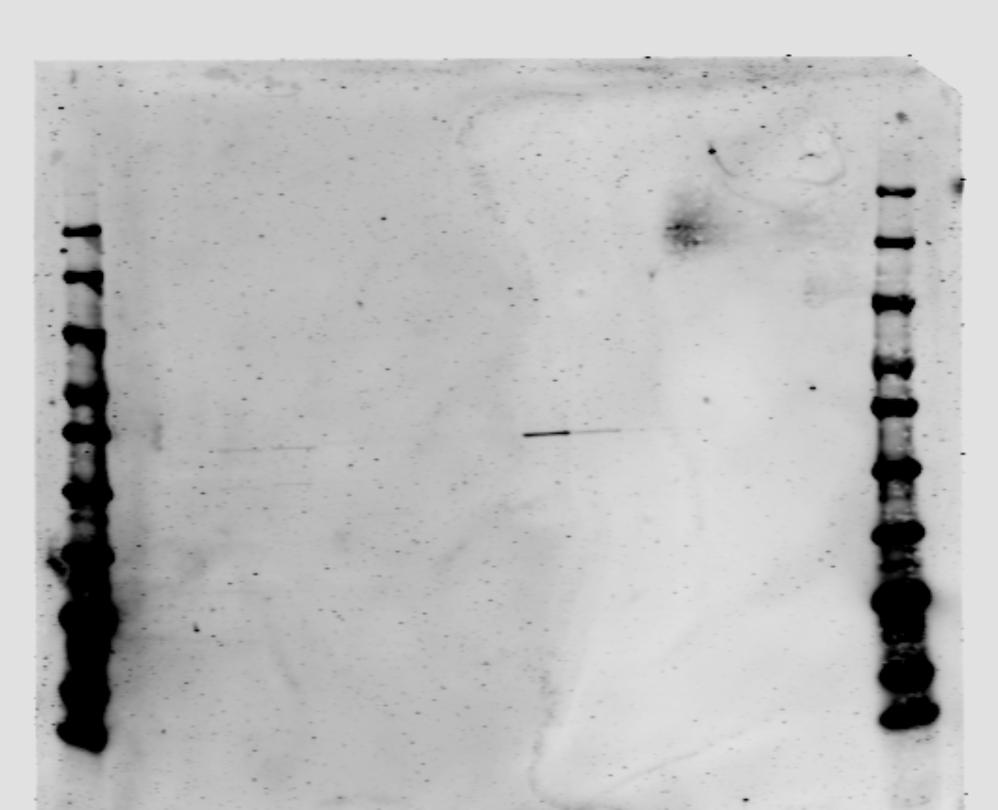

Supplement: Figure 2—source data 2. [file elife-72780-fig2-data2.zip › 97f7cedc-e015-4daa-a946-9c4a0db70777.tif]

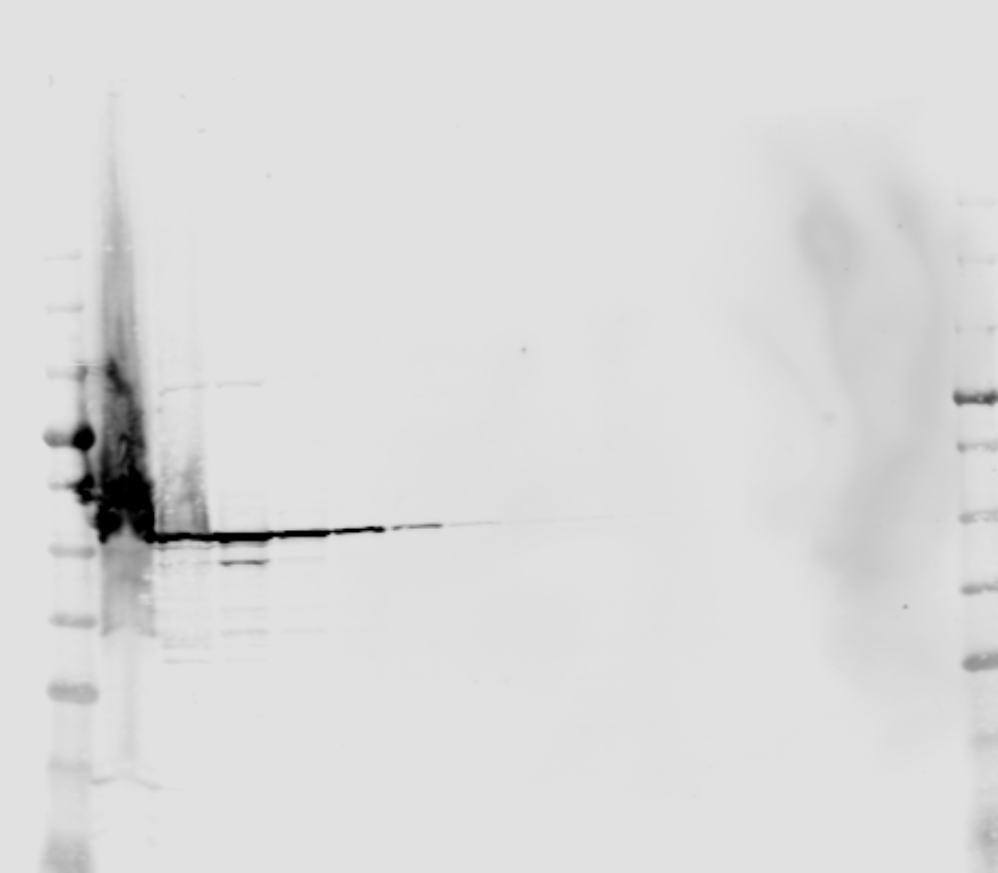

Supplement: Figure 2—source data 2. [file elife-72780-fig2-data2.zip › cee77655-a870-4f0d-80ab-bb5becfa85c9.tif]

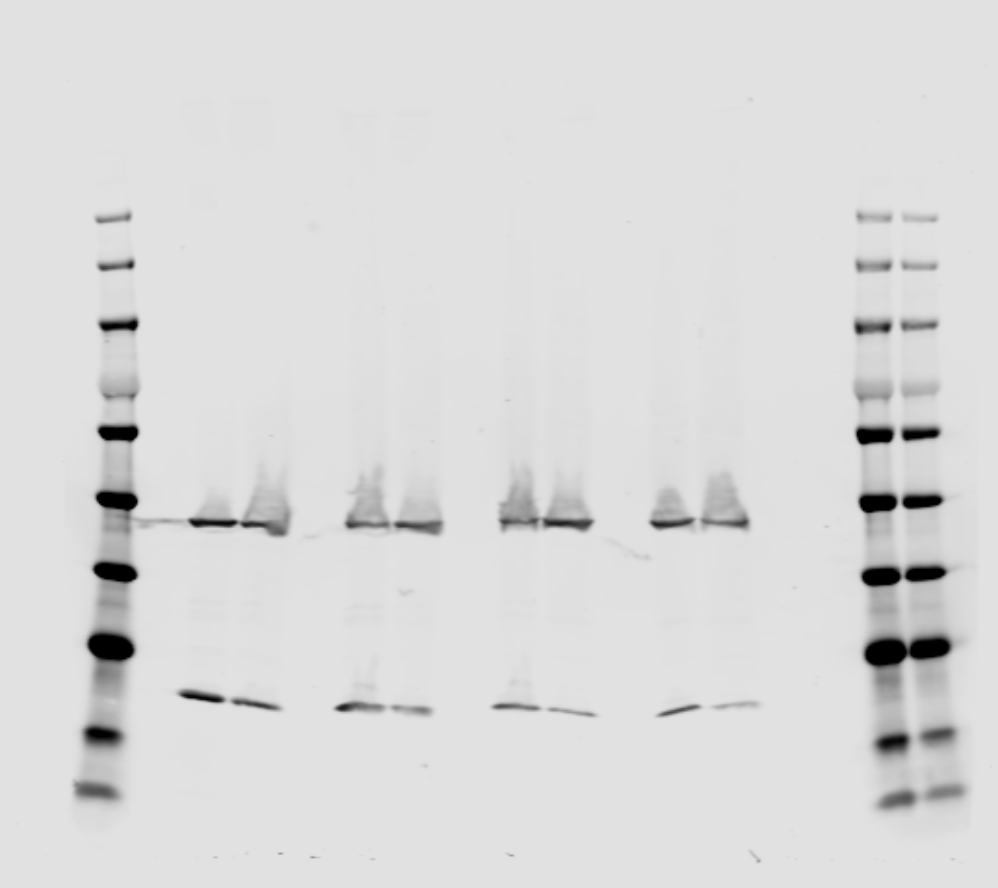

Supplement: Figure 3—figure supplement 1—source data 1. [file elife-72780-fig3-figsupp1-data1.tif]

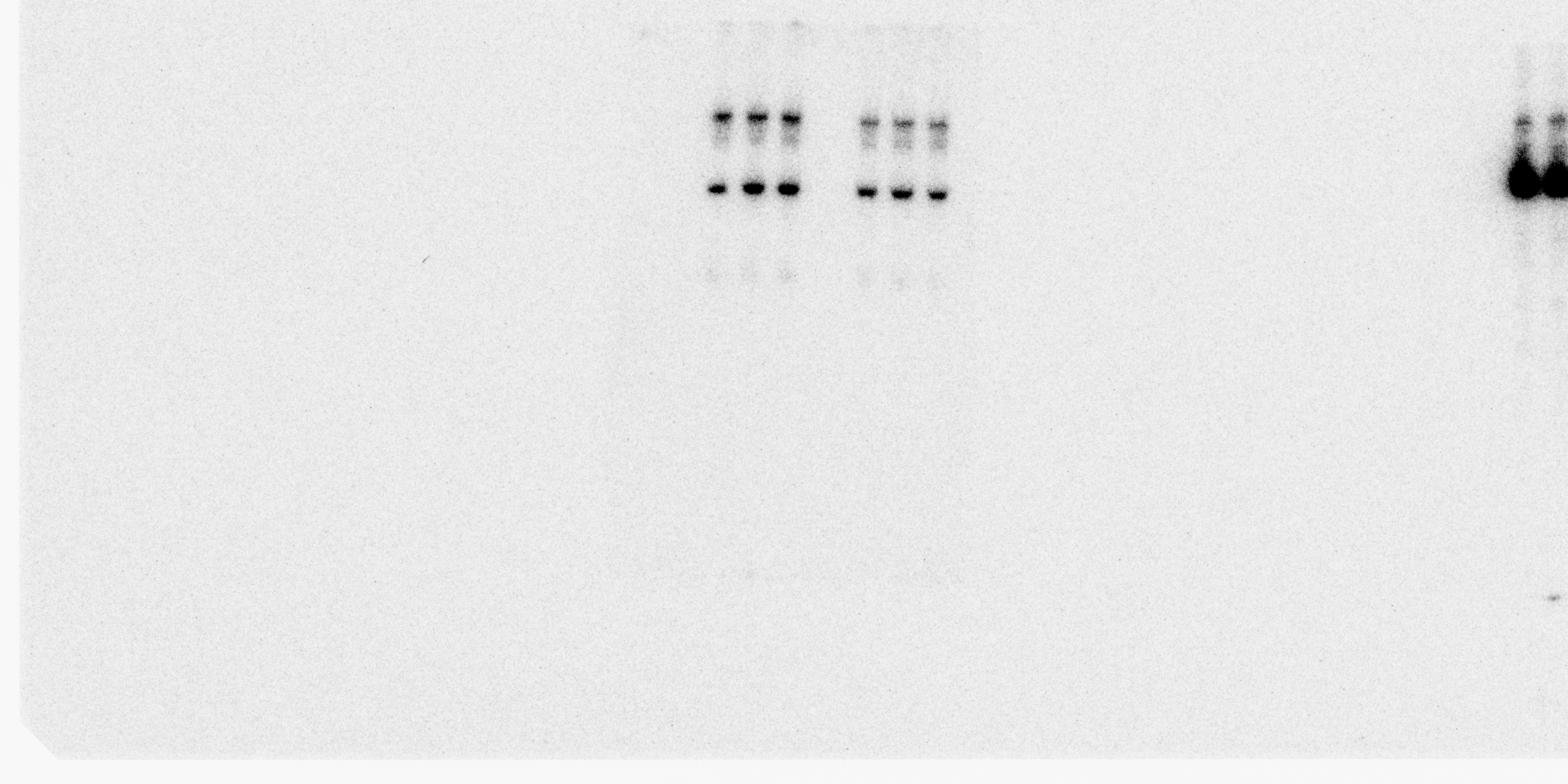

Supplement: Figure 4—figure supplement 2—source data 1. [file elife-72780-fig4-figsupp2-data1.zip › 9ac61bf9-6df3-4607-831a-1d76f22df69c.bmp]

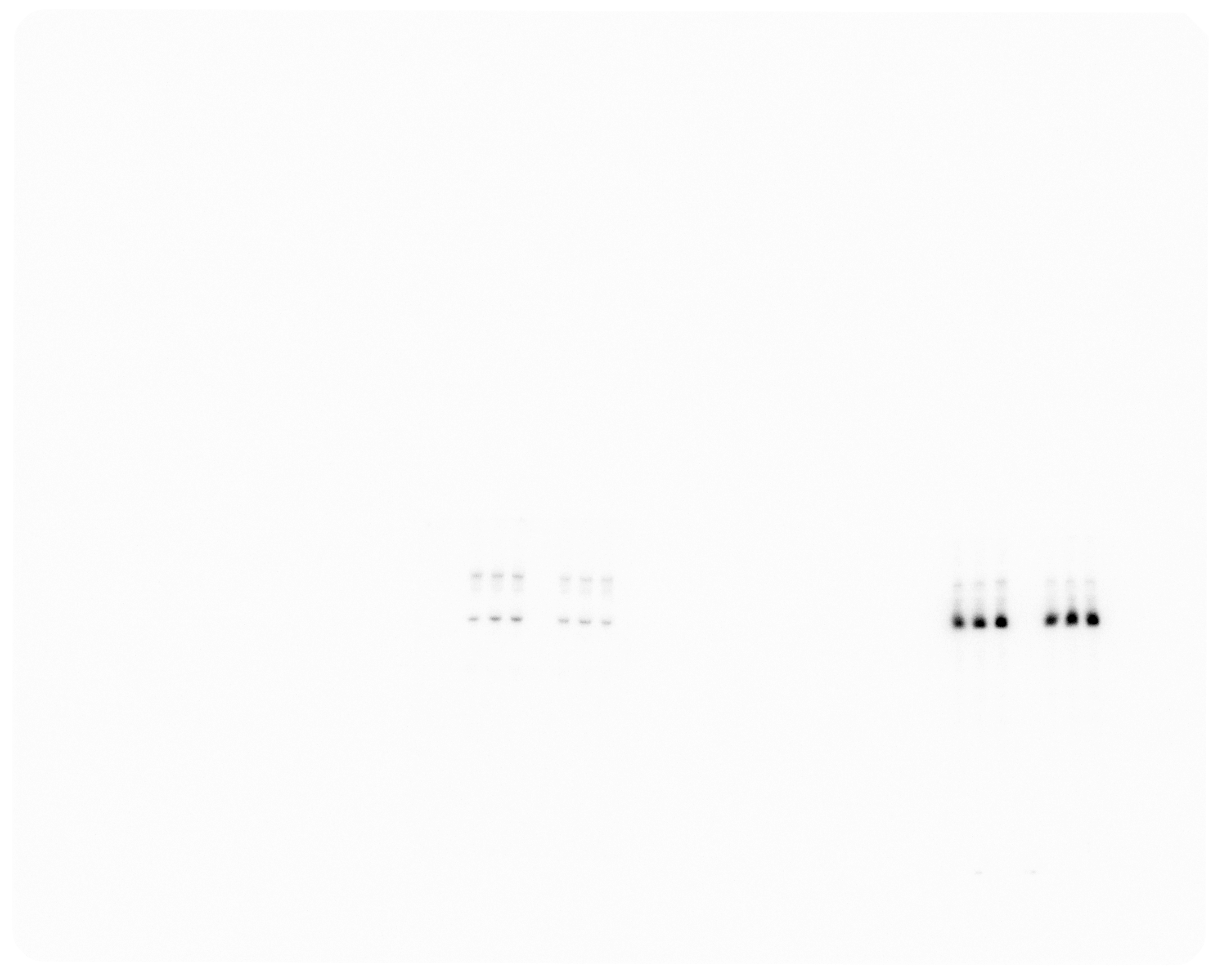

Supplement: Figure 4—figure supplement 2—source data 2. [file elife-72780-fig4-figsupp2-data2.zip › 6e73f650-b87f-4b3c-a6de-055305b0f727.bmp]

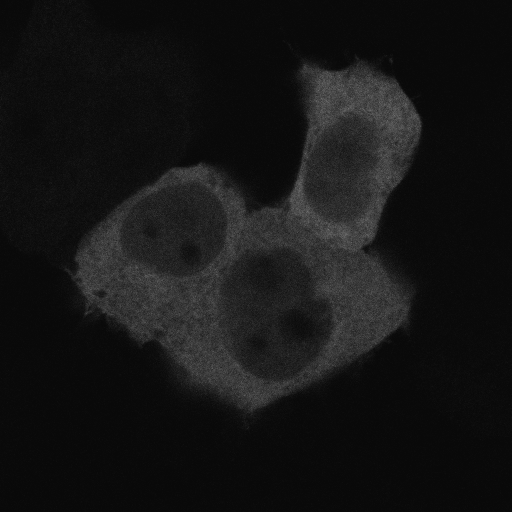

Supplement: Figure 6—source data 1. [file elife-72780-fig6-data1.zip › Figure6A-source data1.tif]

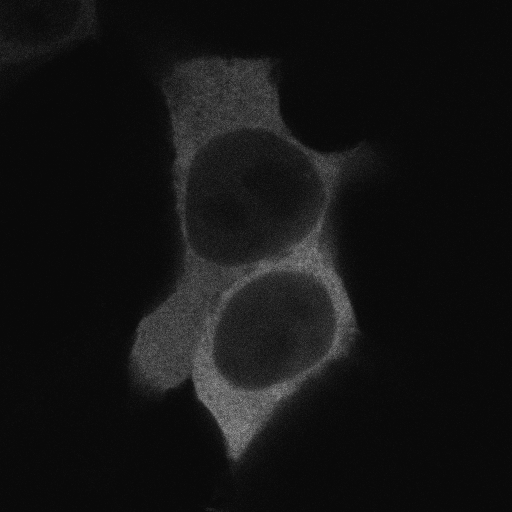

Supplement: Figure 6—source data 1. [file elife-72780-fig6-data1.zip › Figure6A-source data2.tif]

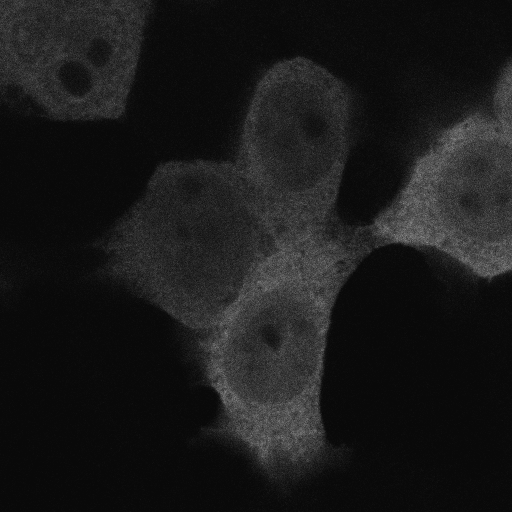

Supplement: Figure 6—source data 1. [file elife-72780-fig6-data1.zip › Figure6A-source data3.tif]

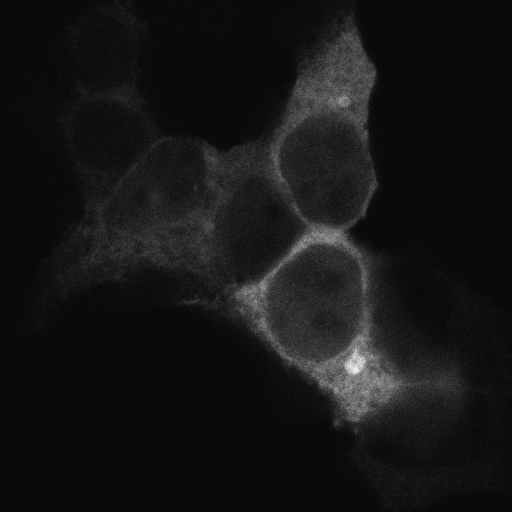

Supplement: Figure 6—source data 1. [file elife-72780-fig6-data1.zip › Figure6A-source data4.tif]

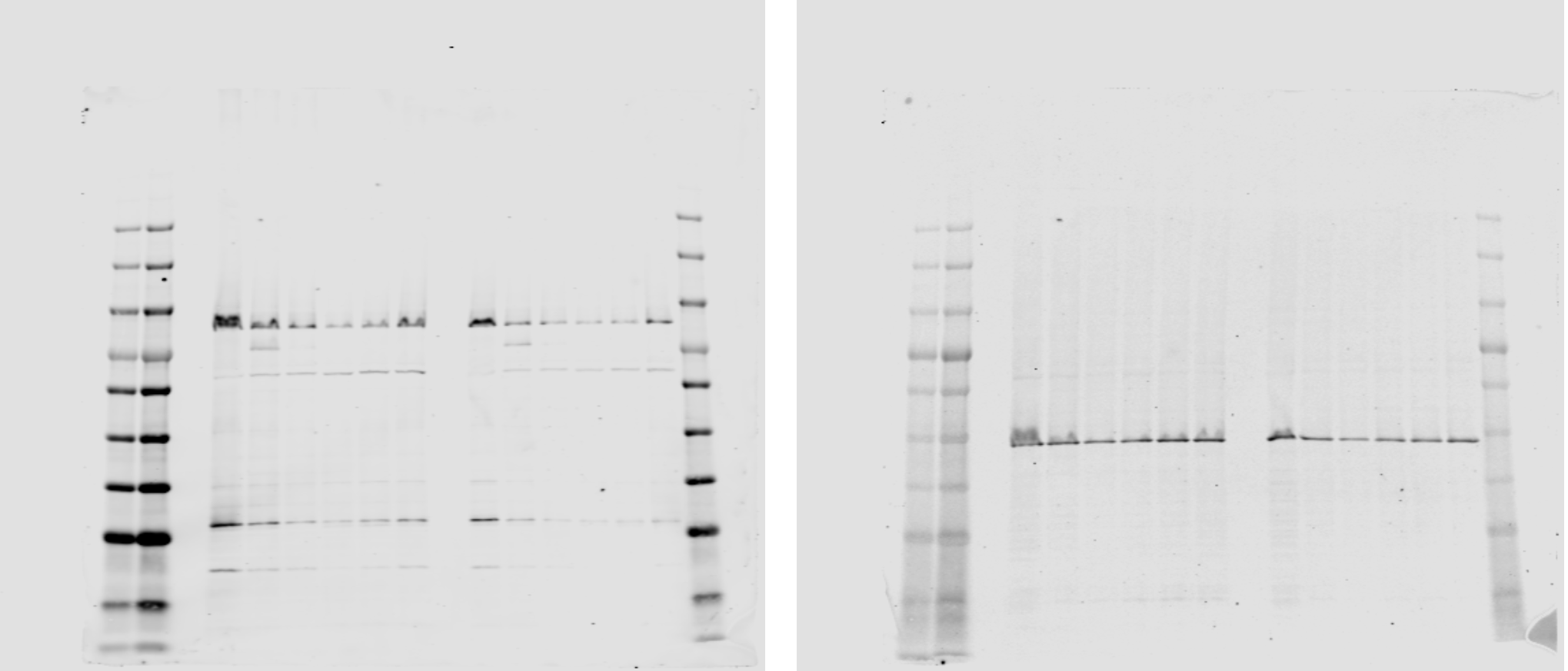

Supplement: Figure 6—source data 2. [file elife-72780-fig6-data2.tif]
